# From novice to expert: agroecological competences of children orphaned by AIDS compared to non-orphans in Benin

**DOI:** 10.1186/1746-4269-7-4

**Published:** 2011-01-10

**Authors:** Rose C Fagbemissi, Lisa L Price

**Affiliations:** 1 Department of Social Sciences, Wageningen University and Research Centre, Wageningen, The Netherlands

## Abstract

**Background:**

AIDS has created new vulnerabilities for rural African households due to prime-age adult mortality and is assumed to lead to impairment of the intergenerational transfer of farming knowledge. There has been scant research to date, however, on the impacts of parental death on farming knowledge of children made orphans by AIDS. The question we investigate is if there is a difference in agricultural expertise between AIDS affected and non-affected adults and children.

**Methods:**

The research was carried out in rural Benin with 77 informants randomly selected according to their AIDS status: 13 affected and 13 non-affected adults; 13 paternal, 13 maternal and 13 double orphans; and 12 non-orphan children. Informants descriptions from pile sorting exercises of maize and cowpea pests were categorized and then aggregated into descriptions based form (morphology) and function (utility) and used to determine whether the moving from novice to expert is impaired by children orphaned by AIDS. Differences and similarities in responses were determined using the Fischer exact test and the Cochran-Mantzel-Haenszel test.

**Results:**

No significant differences were found between AIDS affected and non-affected adults. Results of the study do reveal differences in the use of form and function descriptors among the children. There is a statistically significant difference in the use of form descriptors between one-parent orphans and non-orphans and in descriptors of specific damages to maize. One-parent paternal orphans were exactly like non-affected adults in their 50/50 balanced expertise in the use of both form and function descriptors. One-parent orphans also had the highest number of descriptors used by children overall and these descriptors are spread across the various aspects of the knowledge domain relative to non-orphans.

**Conclusions:**

Rather than a knowledge loss for one-parent orphans, particularly paternal orphans, we believe we are witnessing acceleration into adult knowledge frames. This expertise of one-parent orphans may be a result of a combination of factors deserving further investigation including enhanced hands-on work experience with the food crops in the field and the expertise available from the surviving parent coupled with the value of the food resource to the household.

## Background

AIDS has created a new category of vulnerable rural African household because its impact reduces food production and livelihood viability and creates a spiral of food decline [1-3]. This undermining of rural livelihoods is due to reductions in resources. Two of these farming resources, labor and knowledge, are interlinked in the case of parental death for rural children in Africa [2,4]. Both the loss of productive adult family farm labor and impairment in the intergenerational transfer of traditional food production knowledge are noted as core impacts of the pandemic that lead to more child labor, a greater emphasis on producing to meet food consumption needs, loss of role models for the young to learn from, reduction in the body of traditional knowledge, and loss of the experienced hand of parent as teacher of farming knowledge [4]. Bell et al. [5] project that a breakdown in the transmission of traditional knowledge from one generation to the next leading to an erosion of the stock of human capital can have devastating economic effects and lead to economic collapse of nations severely affected by the HIV pandemic. "The weakening of these transmission processes is insidious; for its effects are felt only over the longer run, as the poor education of children today translates into low productivity of adults a generation hence" ([5], p.8). With the illness and death of one or both parents due to AIDS, children experience multiple life complications. In addition to psychosocial distress caused by the illness and death of one or both parents, they are faced with the family's economic problems, inadequate household food supplies, and taking on new responsibilities as food providers [6-10]. Twelve million children in sub-Saharan Africa are estimated to have lost one or both parents due to AIDS [11].

National and international agencies have shown increasing concern about food security in rural African communities faced with the pandemic [12-14]. Some of the interventions to shore up local food production include support minimizing labor needs, improving harvests, and using local resources to reduce food shortages [1,13-15]. The basic assumption of the food policy and development organizations such as the World Bank and FAO is that traditional knowledge is almost exclusively transmitted from parents to children.

A knowledge based intervention for child/adolescent farmers is the Junior Field and Life Schools covering agricultural knowledge and life skills for orphans and other vulnerable children between the ages of 12 and 18 [16-18]. This knowledge-based approach has its foundation in the well known Farmer Field School approach for adults, and emphasizes learning about agricultural field ecology in a participatory manner [19]. A number of scholars, however, suggest the importance of assessing the impacts of the schools, but there are also indications that needs assessments should be undertaken prior to implementation of such programs [13,14,20-23]. Research in anthropology/ethnobiology has also shown it is difficult to generalize about the loss of knowledge, knowledge gaps, and transmission of environmental and food knowledge and thus indicates that educational interventions could better be supported by empirical studies [20,24,21-31]. This is particularly the case with food and environmental knowledge of children in relation to the adults in their culture [20,25,28,29,31-33].

Knowledge is an element of culture and is key to people doing what makes sense to them [34]. Thus, knowledge is a critical component to the mental models people have that influences their interpretation of the world around them [3,24,35,36]. Knowledge, however, is not static. Individuals constantly use the stimulus they receive from their surrounding environment to build their understandings of the world in a process that in the end generates a set of experiences and perceptions grounded in their culture and particular life situation, which ultimately, shapes expertise. The research findings of Reyes et al. [37] illustrate that different activities exert different effects on knowledge competencies in the same culture group. Activities that demonstrate a greater dependency on forest products being positively correlated with greater knowledge of plants and likewise, activities that are farther removed from forests (such as wage labor) showing individuals having less plant knowledge. Not all activities exert the same effect on knowledge.

In this paper the crop pest knowledge of farming adults and children in the context of AIDS is examined. It is anticipated that AIDS will be associated with differences in life situations that would result in intracultural variation in agroecological knowledge. Also examined is how the parental presence operates when the focus is on the agroecology - in this case knowledge of staple food pests (maize and cowpea). The differences associated with being a child orphaned by AIDS or non-orphan will be investigated, and the effect of being a child orphaned by AIDS or a non-orphan living with one's own biological parent, or having no biological parent at all will be examined. The differences among adults, among children and between adults and children in relationship to being affected by AIDS in the Couffo region of Benin are documented. Complementing an earlier study which examined knowledge differences by focusing on pest naming ability [20], the research reported on in this paper focuses on the various kinds of descriptors that farmers use when talking about pests.

### Intracultural variation of agroecological competence in the context of HIV/AIDS

Intracultural variation in folk biological knowledge has been well-documented [37-39]. Individuals generally vary in their abilities, motivations, and opportunities to learn about living things. While there is contemporary acceptance of intracultural variation, work focused on the nature of folk biological classification and taxonomies has also played a role in the construction of methods to gauge competencies and knowledge. Berlin is well known for the emphasis on morphology in folk classification (form) [40,41]. There has also been the position of Hunn that supports the consideration of the importance of utilitarian factors (function) in how a given human population names and classifies organisms [42]. Boster and Johnson [38], however, have elaborated a compromise position with respect to the foundation of people's rational in folk biology in their study of novice and expert judgments. According to these authors, "humans are purposive beings; their activities and works, including classification systems, have to be understood as outcomes of their intentions" ([38] p.867). They additionally note that curiosity about the natural world also guides people in their acquisition of familiarity with biological diversity. Furthering their position, Boster and Johnson [38] demonstrate the role of both form and function in similarity judgments made by experts (those working with a resource) compared to novices who emphasize form.

In this paper, form (morphology, and related aspects) and function (utility, and related aspects) are used to determine who is novice or expert, and whether the moving from novice to expert is impaired by children being orphaned by AIDS. According to Hunn [42], utilitarian classifications are rooted in patterns of use while morphological information is available to anyone who cares to observe natural organisms. Cultural knowledge of the utility of these organisms usually requires experience and direct communication with those who know.

### HIV/AIDS and agroecological knowledge of child and adult farmers

The main objective of this paper is to present the differences we uncovered in agroecological knowledge between the AIDS affected and non-affected adults and children (orphans and non-orphans), in terms of their use of form and function descriptors in relation to maize and cowpea pests. Consequently, we examine those particular living things labeled by farmers as '*enemy of the crops*' for maize and cowpea. The paper uses the main assumption in ethnoecology, which is based on language as gateway to uncovering knowledge in that people's knowledge and beliefs will emerge through the way they talk about things and categorize them [20,21,36,43]. Language, thought and culture are interlinked and it is expected that there are overarching similarities among the respondents because the population of the study are members of the same culture group living in close proximity, are all farmers, and are speakers of the same language.

It was anticipated that there would be differences based on the impacts HIV has had on the study villages. Death of productive household members means an increase in children's on-farm work and responsibility as well as surviving spouses, mostly women in the study communities, taking over responsibilities previously held by the deceased spouse. It was anticipated that the use value of the agricultural crops increases under the conditions of AIDS where resource decline and enhanced poverty give having a good harvest greater relative importance [1-3,9,10,22,34]. Ultimately, it was expected that AIDS ushered in an element of intra-cultural variation that would be reflected in what people perceive and talk about as conditioned by their experiences.

The enquiry proceeded by comparing child and adult language regarding pests that attack the maize and cowpea crops. The study combines both maize and cowpea pests based on the fact that farmers in the research area often cultivate maize and cowpea in association on the same piece of land.

### Research area

The research was conducted in the Couffo region. This region is located in the southwest of Benin in West Africa. The region has one of the highest HIV prevalence rates in Benin (6% against the national average of 2%). Two representative communalities were chosen as study sites. The study was conducted among the Adja people, the ethnic majority of the Couffo.

The Adja have a reputation in the country as excellent farmers. Some of them also undertake small business activities as a means of additional income generation as well as raising small livestock. The crops present in the Adja landscape are mainly maize (Zea mays, ssp. *Mays*) and cowpea (Vigna unguiculata, ssp. *unguiculata*), followed by tomato and pepper, citrus fruit, teak trees, and cotton [44]. Farming activities involve family labor for food crops, and hired labor for commercial crops. Adults and children are both involved in agricultural activities. Minimizing the agricultural production costs through use of the household labor is common among the Adja. Hence, involving children in farming is quite normal [45].

## Methods

The field research was conducted in June, September and October 2006, and in March 2007. An initial phase consisted of a census of households that experienced the death of one or both parents due to AIDS and the subsequent presence of orphans. These orphans were accordingly characterized as paternal, maternal and double orphans (those who lost both parents). For the purpose of this study participants were randomly selected among each of the six categories of farmers, namely: affected and non-affected adult farmers; non-orphan child farmers (living with both their parents), paternal, maternal and double orphan farmers. AIDS affected adults in this study are adults who lost their partner (wife or husband) or adults who foster orphans in their households and are providing care to them. The non-affected adults are those who have not experienced the loss of household members due to AIDS and were not fostering orphans. Eventually, 77 respondents participated to the study, among whom were 26 adults (13 AIDS affected and 13 non-affected) and 51 children between the ages of ten and fourteen (13 paternal orphans, 13 maternal orphans, 13 double orphans, and 12 non-orphans).

The study consisted of comparing different categories of informants (children and adults, affected and non-affected) farmers views on maize and cowpea pests using their semantics, and documenting differences, with the aim of showing whether AIDS status has any influence on the way the farmers talk about the similarities and differences among the pests [39,46-48]. In the generation of the data, the cultural domain was first captured [49,50]. A free listing procedure was conducted using separate list tasks for maize and for cowpea, which were administered on different days [39,51]. Participants were asked to '*name all living things you think are threats to your maize and cowpea on the farm'*. The free listing was followed by a single pile sort, where informants were asked to put together items generated from the free lists that they thought were similar. They were asked to make as many piles as they liked, but there had to be at least more than one pile [49,50]. These two procedures constituted the entry point of the study. After the pile sort exercise, follow-up conversations were conducted in which farmers were asked to explain as much as they could about the reasoning behind the groupings (the piles) they made. It is especially the outcomes of these follow-up conversations that form the central point of analysis in this article. Thus, these series of dialogues with Adja farmers (children and adults) helped to gather the detailed data that allowed the identification of criteria farmers used in their discussions of pests [38,52,53].

### Analyzing farmers' maize and cowpea pests descriptors

Based on previous work [21,50,38,52,53] the starting point was the use of key words to determine the main content of farmers' semantics. Hence, words that carried the same meaning were put together. The next step consisted of grouping those words/concepts into the categorized descriptors according to the message they conveyed. For instance, one farmer might say that the reason for putting two pests together was that they both *are found on the maize plant *and they *make holes in the maize stem*. These are examples of a farmer describing pests by referring to the habitat (found on maize plant) as well as the specific agronomic damage caused to maize (hole in the stem). These descriptor categories included both morphological and utilitarian groupings for analysis. This exercise was done for all the information collected from the interviews with the farmers. A second level of analysis was to uncover similarities and differences among farmers with respect to the descriptions. Thus, farmers' descriptions were analyzed and differences between the subgroups of farmers examined. Statistically significant differences were assessed using a number of methods. The Fisher's exact test calculates the difference between the data observed and that expected and is appropriate to categorical data where the sample size is small and can be used regardless of the sample characteristics (non-parametric). To this end, the Fisher's Exact test is based on testing the alternative hypothesis H1: P_1 _# P_2_, as opposed to the null hypothesis H0: P_1 _= P_2 _(*no differences among groups of informants*). P_1 _and P_2 _represent the probability of an individual of a given subgroup of farmers (categories) using a given descriptor to express their perceptions about maize and cowpea pests.

The next step was to examine how much these observed differences in descriptions translated into differences in expertise among farmers. To this end, the descriptors were re-grouped into two major categories: on one hand there are characteristics linked to the form of the living things (kind of pests; morphology and locomotion), and on the other hand there are descriptors linked to the functional characteristics (agronomic aspects; management aspects and utility) [38]. Differences were examined by AIDS affection status and generation.

The overall combined effect of AIDS on respondents' perceptions was examined using the Cochran-Mantzel-Haenszel test [54,55]. This test determined whether there was a significant association between the descriptor used by a respondent given the fact that this person is a child or an adult, while adjusting for the effect of AIDS affection. The significance of the association depends on that of the odds ratio, and is reported using the conditional independence statistics. This statistic has three components: the Cochran-Mantel-Haenszel's (CMH) assumes a common odds ratio and tests the null hypothesis that the variables X (generation - child/adult) and Y (descriptors - form/function) are conditionally independent, given Z (AIDS affection); the Mantel-Haenszel (MH) test, which measures the strength of association between the variables by estimating their odds ratio for a 2 × 2 × 2 contingency table, and Breslow-Day (B-D) statistic, which tests the homogeneity of the odds ratio. When CMH p-value is high, this means that the variables 'generation' and 'descriptor' are conditionally independent, given 'AIDS affection'. If the p-value for B-D test is high, it is possible to summarize their conditional association by a single odds ratio, which means there is a homogeneous association between the AIDS status and generation. In the end, when the null hypothesis for the M-H test is accepted, the analysis of differences is done using 2 separate 2 × 2 contingency tables for X (generation) and Z (AIDS status) with respect to Y (types of descriptors).

### The novice-expert test

Based on Boster and Johnson's findings on novice versus expert judgment of similarity [38], it was assumed that an expert is a respondent who has a balance of form and function with respect to the descriptors they use. From an analytical standpoint, it is anticipated that superior expertise would result in a 50/50 ratio in the combined ability of respondents to use form and function descriptors. In this regard, an index of expertise (IE) was calculated by checking the number of mentions within a group of informants by the total number of items in each category, that is for form and function separately [49,56]. The quality of expertise is obtained by weighting group mean for form and function respectively, by the sum of means for form and function. This ratio was eventually used to map the distribution of respondent's expertise for form and function for each group of respondents [57]. The ratios of form-function distribution were then submitted to Fisher's exact test to check for the differences between the groups of respondents.

## Results

Eight types of descriptors were extracted from the conversations with farmers that followed the pile sorting of maize and cowpea pests. Most numerous were descriptors of agronomic damages caused by the pests (generic damages as well as the one specific either to maize or to cowpea). The generic agronomic damages descriptors reflect the fact that there are pests that equally attack maize and cowpea according to farmers, causing similar damages on the two crops. Specific agronomic descriptors express that the pests are found or feed on one of the crops, not the other. For example, farmers use words such as '*remove seed from the soil' or 'cut the seedlings' *to reflect generic damages, and '*attack maize cobs' *or '*make holes in the cowpea pods' *for damages specific to maize or cowpea respectively. In addition, there are also aspects linked to the habitat or ecology of the reported pests (Table 1).

**Table 1:** Categorizes descriptors of maize and cowpea pests elicited after pile sorting exercises

*Descriptors category*	*Farmers' words*
1. Kind of pest	they are domestic mammals; they are birds; they are wild animals

2. Morphology & locomotion	shape: have different shapes; have hairs or not; have a wavy body; have paws or not; have wings or not; have legs; size: big, small; invisible (they are only noticeable by their damages); motion: walk; jump color: red; green; white; black

Agronomic aspects:	

3. habitat/ecology	live on the farm, live in the soil, find on maize, find on cowpea, find on leaves, find on stems, stay on the apex, live in maize cobs, find in maize grain, live on cowpea leaves, find in cowpea pods, find on cowpea grains.

4. generic damages	very dangerous for the plants, come in flocks, remove seed from soil, eat seeds, eat seed germ, block seed germination, eat seedlings, attack/cut plant roots, uproot seedlings, cut seedlings, cut plants, eat stems, make holes in the leaves, eat the leaves, remove the grains, eat the grains, make holes in the grains.

5. specific damages to maize	eat maize seeds, cut maize seedlings, eat maize leaves, cut maize plant, uproot maize plants, suck maize stems, eat maize stems, cut maize leaves, attack maize cobs, make hole in maize cobs, eat maize cobs, eat maize grains, make holes in maize grains.

6. specific damages to cowpea	cover cowpea plants, attack cowpea plants, destroy cowpea plants, stop cowpea growth, eat cowpea stems, cover cowpea stems, eat cowpea leaves, make holes in cowpea leaves, cause cowpea leaf loss, twist cowpea leaves, cut cowpea leaves, cover cowpea leaves, cover cowpea plant apex, cut cowpea flowers, make holes in cowpea pods, eat cowpea grains.

Management and utilization aspects	

7. Managing the pests	Easy to kill, they are troublesome, difficult to fight, need the use of insecticide, resistant to insecticide, no need of insecticide.

8. utility	We sell them; we eat them; they are our poultry

Number of respondents (N) = 77	

### AIDS affection and farmers perception of pests among child and adult farmers

There are, in general, two significant differences between orphan and non-orphan child farmers (Table 2). These are the differences in the use of generic descriptors (p-value 0.05), and damage to maize descriptors (p-value 0.05). In the case of both of these descriptors, non-orphans mentioned more than orphans.

**Table 2:** Percentage of mentions of each descriptor by respondent group as a portion of that groups total mentions and results of the Fisher exact test applied to number of mentions by informant group.

	*Descriptors*										
	
*Respondents groups* *N = 77*	Kind of pest	Morphology/locomotion	Form attributes	Agronomic aspects				Pest management	Utility	Functional attributes	Total
								
				Habitat/ecology	Generic damages	Specific damages to maize	Specific damages to cowpea				
One parent orphans vs. double orphans											

Orphans with own parent	6	8	14	7	17	21	20	7	14	86	100
Double orphans	5	15	20	5	23	15	23	0	10	80	100
Fisher exact test (*p-value*) ^b^	0.13	0.14	0.09	0.33	0.18	0.14	0.15	0.05*	0.34	0.09	

One parent orphans vs. non orphans											

Orphans with own parent	6	8	14	7	17	21	20	7	14	86	100
Non orphans	0	6	6	3	31	31	29	0	0	94	100
Fisher exact test (*p-value*)	0.13	0.04*	0.02*	0.19	0.13	0.02*	0.15	0.24	0.22	0.02*	

Double orphans vs. non orphans											

Double orphans	5	15	20	5	23	15	28	0	10	80	100
Non orphans	0	6	6	3	31	31	29	0	0	94	100
Fisher exact test (*p-value*)	0.26	0.11	0.02*	0.41	0.27	0.05*	0.5	n.d ^c^	0.32	0.02*	

	***Descriptors***										
	
	Kind of pest	Morphology/locomotion	**Form attributes**	Agronomic aspects				Pest manage-ment	Utility	**Functional attributes**	Total
								
				Habitat/ecology	Generic damages	Specific damages to maize	Specific damages to cowpea				

Overall effect of AIDS affection among the children: orphans vs. non orphans											

Orphans	6	11	17	6	18	18	23	5	13	83	100
Non orphans	0	6	6	3	31	31	29	0	0	94	100
Fisher exact test (*p-value*)	0.13	0.17	0.04*	0.29	0.05*	0.05*	0.03	0.18	0.25	0.02*	

Effect of AIDS affection within generation: children vs. adults											

Children	5	10	15	5	21	21	24	4	10	85	100
Adults	3	11	14	8	24	22	29	3	0	86	100
Fisher exact test (*p-value*)	0.27	0.37	0.43	0.29	0.21	0.25	0.21	0.32	0.32	0.48	

a = % of mentions of each type of descriptor out of total mentions by respondent group; b = Fischer exact test significance level as: *p = ≤0.05; c = there is no difference.

Looking in greater detail at the categories of orphans it can be seen that there are some differences based on the results of the Fisher's exact test between the one-parent orphans and double orphans as well as both of these kinds of orphans relative to non-orphans (Table 2). One-parent orphans used the descriptors of fighting the pests which no double orphans used (p-value 0.05). There is a statistically significant difference in the use of morphological descriptors (p-value 0.04), and in the use of descriptors of specific damages to maize (p-value 0.02) between one-parent orphans and non-orphans. One-parent orphans used more morphological descriptors and fewer maize damage descriptors relative to non-orphans. Results from the Fisher's Exact tests depicted in Table 2 also reveal that non orphans show a difference from double orphans, as well as with one-parent orphans regarding their use of descriptors of damages caused on maize by pests (p-value 0.05 respectively).

One-parent orphans have the highest mean number of descriptors per child (3.19) compared to double orphans (3.07) and non-orphans (2.66). The frequency of responses (seen in Table 2) also illustrates that orphans have their mentions spread across all the descriptors and thus through several aspects of the agroecological knowledge, while non-orphans mentioned descriptors in fewer categories. Notably, non-orphans had no mentions of utility (use value) of the pests (those such as birds that can be eaten) nor did they mention aspects of pest management.

Table 3 provides deeper insight into the combined effects of AIDS and generation. The results presented in this table are the outcomes of the Mantel-Haenszel test of partial independence between AIDS status (i.e. affected/non- affected) and generation (i.e. child/adult).

**Table 3:** Testing the combined effect of generation and HIV/AIDS on farmers' cultural expertise in the Couffo given the percentages of mentions of form and function descriptors.

	***Descriptors ***^**a**^ (%)	
	
*Group of respondents*	Form	Function
Orphans	17	83
Non orphans	6	94
Affected adults	11	89
Non-affected adults	17	83

**Test of association:**		

B-D's homogeneity of the odds ratio ^b^	0.66 (ns)	0.08 (ns)
M-H conditional independence ^c^	0.05*	0.05*
Estimate of the odds ratio	2.97	1.399
*p*-value (2-sided)	0.05*	0.09 (ns)
	
95% confidence interval (CI)	[0.6 - 6.5]	[0.5 - 3.8]

Number of respondents N = 77		

a = % of mention within each category of descriptors; b = Breslow-Day test significance;

c = Mantel-Haenszel test, with * = p ≤ 0.05 and ns = not significant.

In the first half of the table, the descriptive statistics show that non-affected adults and orphans have the same distribution of form and function descriptors of pests, while non-orphans use function descriptors more (see also Table 2). In the second half of Table 3, the Breslow-Day test of the odds ratio shows a statistically non-significant value, which means that the value of the odds ratio can be used to interpret the internal variations in the use of descriptors among the respondents, that is, their expertise. Thus, the respective estimate of the odds ratio, which is 2.97 for form descriptors and 1.399 for function ones, shows that there is almost three times the likelihood for a child, if affected by AIDS (that is, orphan) to mention a form descriptor compared to the other respondents. There is also almost 1.5 times greater likelihood that an orphan mentions a function-related attribute of pests.

A closer look at the first part of Table 3 (and the results presented in Table 2) on the differences in the use of form and function among the respondents shows that Adja farmers, whether affected by AIDS or not, seem to all use functional descriptors which is the primary indicator of expertise in a cultural group. Hence, the point is the examination of the quality of their expertise to gain more insights on the value of this expertise and the related intracultural distribution of the agroecological knowledge among the respondents. Table 4 and 5 show the results of a further analysis of the differences in expertise.

**Table 4:** Distribution of the quality of expertise among Adja farmers given the ratios of form and function of their aggregated index of expertise, and the Fisher exact test for the ratios.

	***Descriptors ***^**a**^ (%)	
	
*Group of respondents* (N = 77)	Form	Function
Adults and children		

Children	40	60
Adults	41	59
*p-value*	0.17 (ns)	0.13 (ns) ^b^

Children (n = 51)		

Orphans	45	55
Non-orphans	22	78

*p-value*	0.05*	0.05* (ns)

Adults (n = 26)		

affected	45	55
non-affected	50	50
*p-value*	0.36 (ns)	0.08 (ns)

a = Values represent weighted proportions of form and function in the indexes of expertise; b = Fisher exact significance, with * = p ≤ 0.05 and ns = not significant.

**Table 5:** Distribution of the quality of expertise among child farmers given the ratios of form and function of their aggregated index of expertise, and the Fisher exact test for the ratios.

	***Descriptors ***^**a**^ (%)	
	
*Group of respondents* (N = 51)	Form	Function
One parent orphans (n = 26)		

Paternal	50	50
Maternal	30	70
*p-value*	0.15 (ns)	0.28 (ns)

One parent orphans/no parent (n = 39)		

Paternal/maternal	40	60
Double orphans	58	42
*p-value*	0.09 (ns)	0.05*

No parent/two parents (n = 25)		

Double orphans	58	42
Non-orphans	22	78
*p-value*	0.05*	0.07 (ns)

a = Values represent weighted proportions of the indexes of expertise;

b = Fisher exact significance, with * = p ≤ 0.05 and ns = not significant.

The analysis of respondent expertise in the use of descriptors aggregated into form and function showed no statistically significant difference between adults and children (Table 4).

The results of the Fisher's exact test of the index of expertise for form and function, however, do show a significant and inverted difference between orphans and non-orphans (at the 0.05 level). Non-orphans used significantly fewer descriptors of form and significantly more of function than orphans (see Table 4). From Table 4 it appears that globally, orphans and affected adults had a better expertise with respect to their ability to use a balance of form and function to reflect on their perceptions about maize and cowpea pests. In fact, their ratio of form-function expertise was 45/55, which was close to the 50/50 that was anticipated.

The differences in expertise for the use of form and function descriptors between orphans and non-orphans were further investigated by disaggregating the category of orphans (Table 5). A significant difference was found in expertise for the use of function descriptors between one-parent orphans and double orphans in the use of form descriptors (p-value 0.05). A difference was also found in expertise between double and non-orphans (p-value 0.05) for the use of form.

The examination of the ratio form-function in each group shows, first, that there is no significant difference between AIDS affected and non-affected adults, and second, that double orphans rely more on form and less on function relative to the other children that still have at least one of their parents. Precisely, and in the light of the results in Table 4 and 5, it appears that children orphaned by AIDS, and especially one-parent orphans, have a more balanced expertise in the use of form and function descriptors for crop pesta. The distribution of the agroecological expertise, as reflected in the ratio of form-function for non-affected adult farmers shows the closest expertise (a split of 50/50) to that of the orphans (a split of 45/55), and is identical to that of paternal orphans (a split of 50/50) (see Table 4 and 5). This information, combined with the findings in Table 3 indicates that the combined effect of AIDS and generation on expertise is one of HIV and AIDS hastening the acquisition of agroecological knowledge among children orphaned by AIDS in the Couffo.

## Discussion and conclusion

This study examined the differences between AIDS affected and non-affected adults and children in the way they describe pest problems in maize and cowpea. No significant difference between AIDS affected and non-affected adults was discovered nor were there differences overall between adults and children (irrespective of AIDS status). There are, however, some areas of statistically significant differences in the kinds of descriptors AIDS affected farm children and non-affected farm children use, falling into the categories of "form" which was based on morphology and locomotion and "function" that included habitat and ecology, generic and crop specific damages caused by pests, pest management, and utility (use value for e.g. consumption). Ultimately, there is a statistically significant difference in the use of form and function, and the respective expertise attached to their use, between non-orphans and orphans with non-orphans using form less and function more. However, orphans, like adults, have a more balanced expertise in the use of form and function with fatherless orphans having a balance of 50/50 in the descriptors they used, exactly like non-affected adults.

These differences between being more of a novice or more of an expert [38] are potentially related to how much the children interact with an adult and the quality of that interaction. Importantly, we suggest the children's level of interaction with the resources/items under study was vital to the differences uncovered [20,37]. Our earlier study supports the finding that children who remain with the surviving parent (mother) have more expertise compared to other children [20]. The results show, however, that there are no novices in the strictest sense, that is, totally dependent on visual cues only from observations of form. Both form and function were present in the descriptors of both AIDS affected and non-affected adults and children. The degree to which each kind of descriptor was present in the responses is of particular interest.

In their study, Boster and Johnson [38] found that experts are actually intermediate between the two models of judgment, using form and function aspects fairly equally. In connection to this, an important point to highlight is the observed difference in expertise in evidence between fatherless orphans and other categories of children. It appears that in this study, the child farmers with the most adult-like expertise, that is, the paternal orphans (i.e. those living with a surviving mother), did not abandon early models of understandings of the living things for later ones, but they seem to have accumulated alternative models. This is in accordance with the position defended earlier by Boster and Johnson [38] who argued that novices become experts by starting with readily available models, which are generally superficial (e.g., morphologically based) and gradually acquiring the more abstract ones (e.g., functionally based). There are indications that a similar pattern exists here. Paternal orphans have been shown to be more engaged in farming through the application of their own labor in the fields, and thus have the opportunity to gain more in-field expertise and make more observations, see [58,59]. These results reflect paternal orphans having new responsibilities, conducting more work in the agricultural fields, and having one surviving parent to obtain needed knowledge from [60,61] Rather than a knowledge loss for paternal AIDS orphans, it appears there is an acceleration into adult knowledge frames.

Ethnobiological knowledge and practices within any culture vary according to people's social status and context, relations and social networks, income, age and gender, among other attributes [62,63]. The enculturation of children as young farmers is affected by the combined effect of specific parent-child relationships and the type or level of involvement in farming activities. These dimensions have important implications for gaining expertise in agroecological knowledge. In addition to having one biological parent, selected children probably have more tactical knowledge from engaging in the farm activities [29]. Furthermore, the present study highlights the importance of parent as teacher and role-model to farm household children's acquisition of agroecological knowledge and expertise. Kadiyala and Gillespie [64] found that AIDS has not only deepened vulnerability, but has also impacted the capacity of the extended family to accurately respond to the challenges posed by the pandemic to farm households. This study furthers this observation by showing that foster families appear to not be the best channels for environmental knowledge transfer to double orphans. In this study, double orphans were being raised mainly by grandparents. In addition, some of the double orphans farmed on their own [see 20]. Richter [61] shows that children become more vulnerable when they are cared for by aged relatives due to the conditions of mutual dependency that often exist between adult and child. This mutual dependency could also jeopardize parenting and thus, the acquisition of the agroecological knowledge by double orphans.

The situation of AIDS affected orphan child farmers is one of a mixture of vulnerability and agency, dependency and being depended upon. These findings indicate that rather than a loss of agroecological knowledge, there may be a strengthening of knowledge among selected children who happen to be orphans. Thus, these findings indicate a need to rethink the issue of farming knowledge and its loss in AIDS affected communities.

## Competing interests

The authors declare that they have no competing interests.

## Authors' contributions

RF collected the data and analyzed the data under the supervision of LLP. RF and LLP interpreted the data and drafted the framework for the paper and the discussion and conclusions. The writing of the article was a joint enterprise. All authors read and approved the final manuscript.
